# Simulating the Effect of Spectroscopic MRI as a Metric for Radiation Therapy Planning in Patients with Glioblastoma

**DOI:** 10.18383/j.tom.2016.00187

**Published:** 2016-12

**Authors:** J. Scott Cordova, Shravan Kandula, Saumya Gurbani, Jim Zhong, Mital Tejani, Oluwatosin Kayode, Kirtesh Patel, Roshan Prabhu, Eduard Schreibmann, Ian Crocker, Chad A. Holder, Hyunsuk Shim, Hui-Kuo Shu

**Affiliations:** 1 Department of Radiology and Imaging Sciences, Emory University School of Medicine, Atlanta, Georgia;; 2 Department of Radiation Oncology, Emory University School of Medicine, Atlanta, Georgia;; 3 Florida Hospital Medical Group, Radiation Oncology Associates, Orlando, Florida;; 4 SE Radiation Oncology Group, Levine Cancer Institute, Charlotte, North Carolina;; 5 Winship Cancer Institute, Atlanta, Georgia; and; 6 Department of Biomedical Engineering, GA Institute of Technology, Atlanta, Georgia

**Keywords:** glioblastoma, magnetic resonance spectroscopy, spectroscopic MRI, radiation therapy planning, metabolic regions-of-interest

## Abstract

Due to glioblastoma's infiltrative nature, an optimal radiation therapy (RT) plan requires targeting infiltration not identified by anatomical magnetic resonance imaging (MRI). Here, high-resolution, whole-brain spectroscopic MRI (sMRI) is used to describe tumor infiltration alongside anatomical MRI and simulate the degree to which it modifies RT target planning. In 11 patients with glioblastoma, data from preRT sMRI scans were processed to give high-resolution, whole-brain metabolite maps normalized by contralateral white matter. Maps depicting choline to N-Acetylaspartate (Cho/NAA) ratios were registered to contrast-enhanced T1-weighted RT planning MRI for each patient. Volumes depicting metabolic abnormalities (1.5-, 1.75-, and 2.0-fold increases in Cho/NAA ratios) were compared with conventional target volumes and contrast-enhancing tumor at recurrence. sMRI-modified RT plans were generated to evaluate target volume coverage and organ-at-risk dose constraints. Conventional clinical target volumes and Cho/NAA abnormalities identified significantly different regions of microscopic infiltration with substantial Cho/NAA abnormalities falling outside of the conventional 60 Gy isodose line (41.1, 22.2, and 12.7 cm^3^, respectively). Clinical target volumes using Cho/NAA thresholds exhibited significantly higher coverage of contrast enhancement at recurrence on average (92.4%, 90.5%, and 88.6%, respectively) than conventional plans (82.5%). sMRI-based plans targeting tumor infiltration met planning objectives in all cases with no significant change in target coverage. In 2 cases, the sMRI-modified plan exhibited better coverage of contrast-enhancing tumor at recurrence than the original plan. Integration of the high-resolution, whole-brain sMRI into RT planning is feasible, resulting in RT target volumes that can effectively target tumor infiltration while adhering to conventional constraints.

## Introduction

Optimal targeting of glioblastoma (GBM) for radiation therapy (RT) using computed tomography (CT) and magnetic resonance imaging (MRI) remains challenging because of the tumor's infiltrative nature. Traditionally, 2 RT target volumes are created on the planning CT as follows: one defining enhancing tissue and the resection cavity on contrast-enhanced T1-weighted (CE-T1w) MRI and another defining signal changes on a T2-weighted, fluid-attenuated inversion recovery (FLAIR) sequence. However, histological analyses indicate that these sequences fail to capture the true extent of disease because of infiltrative, nonenhancing tumor (1–5). In addition, anatomic MRI provides limited information on the physiological state and biochemical activity of a viable tumor (4). The inability to accurately identify active tumor using conventional neuroimaging remains a limiting factor in GBM RT planning.

Proton magnetic resonance spectroscopic imaging (MRSI) is a molecular imaging technique that maps the metabolism of native small molecules to tumor regions in vivo without exogenous tracers (6, 7). Studies have shown that the addition of magnetic resonance spectroscopy (MRS) to tumor targeting allows identification of unique metabolic regions of interest with significant clinical consequence (ie, regions of tumor progression) (8–12). Moreover, recently, it was shown that the choline to N-Acetylaspartate ratio (Cho/NAA), a marker describing glial cell proliferation, correlates with tumor infiltration, progression-free survival, and contrast enhancement at recurrence in GBM (13). Thus, Cho/NAA regions of interest deserve consideration during target planning, and may be of use if effectively integrated into image guidance.

However, technical limitations have hindered the use of MRS in RT planning. Sequence-specific limitations including poor spatial resolution and limited brain coverage obscure the margins of metabolic abnormalities and prohibit visualization of infiltration into the surrounding brain tissue. This prevents accurate and complete delineation of tumor infiltration, curbing the value of metabolic data in target planning for GBM. In addition, there is a paucity of clinical decision support software for the analysis, visualization, and management of spectroscopic data. Consequently, spectroscopic processing requires a skilled user to evaluate spectra and manually transfer data between platforms, resulting in a complex workflow that is impractical for clinical RT. To address these limitations, we have developed an image acquisition and processing pipeline based on previously established state-of-the-art, high-resolution spectroscopic MRI (sMRI) and automated analysis tools to allow the addition of whole-brain metabolite maps to RT planning (14). Herein, we describe this sMRI workflow, evaluate its integration into RT planning, and determine the degree to which it modifies RT target volumes in GBM.

## Methodology

### Patients

Newly diagnosed, pathologically-confirmed patients with GBM were enrolled in an institutional review board-approved clinical trial. All patients underwent an sMRI scan ≤7 days before standard-of-care chemoradiation, which consisted of a 60 Gy dose (30 fractions of 2 Gy) with concomitant and adjuvant temozolomide. Patients were scanned following surgery (eg, resection or biopsy) and before starting chemoradiation. Patients were at least 18 years of age, had a Karnofsky Performance Status score of ≥70, and were both willing and able to sign informed consent forms. Patients with MRI-incompatible implants, medical conditions compromising RT tolerance, or receiving investigational agents were excluded.

### sMRI Data Acquisition and Processing

Whole-brain, 3-dimensional echo-planar spectroscopic imaging was conducted on a 3 T MRI scanner with a 32-channel head coil array (TIM/TRIO, Siemens Medical Solutions, Erlangen, Germany). The head coil was securely attached and padded with foam blocks to reduce patient motion. Within the Syngo software, first-order shims were manually optimized to a waterline width of ≤25 Hz, and lipid signal was nulled using outer volume suppression with manually placed saturation bands. Tissue water signal was collected in an interleaved manner with the metabolite data for signal normalization and image registration. The sequence time was 17 minutes, plus an additional 2 minutes for manual shimming. Spectroscopic data were processed using the Metabolite Imaging and Data Analysis System (MIDAS) to obtain maps with a nominal voxel size of 4.4 × 4.4 × 5.6 mm^3^ at acquisition; postprocessing yields an effective voxel volume of 1 mL (14, 15). Metabolite maps included choline, creatine, N-Acetylaspartate, and Cho/NAA. These maps were imported into VelocityAI (Varian Medical Systems) for registration to the CE-T1w and T2-weighted/FLAIR images and upsampled into the clinical MRI image space using trilinear interpolation (16). As metabolite levels fluctuate with age, gender, and location, signal standardization was accomplished by scaling maps using the mean metabolite value of contralateral normal-appearing white matter to generate fold-normal(-fold) measures of metabolic abnormality (14). Throughout this paper, “Cho/NAA values” refers to the fold-increase of the ratio as compared with the mean ratio in normal-appearing white matter.

### Conventional and sMRI Target Volume Definitions

Conventional treatment volumes were generated using VelocityAI by radiation oncologists (SK and HKS). Gross target volumes (GTVs) 1 and 2 defined the T2-weighted/FLAIR abnormality and contrast-enhancing tissue/resection cavity, respectively, whereas clinical target volumes (CTVs) 1 and 2 defined these GTVs with 0.7- and 0.5-cm modified margins, respectively. The planning target volumes (PTVs) 1 and 2 consisted of the CTV1 and CTV2 with 0.3-cm margins, respectively. The PTV1 was prescribed to 51 Gy (10 patients) or 54 Gy (1 patient) in 30 fractions. PTV2 was prescribed to 60 Gy in 30 fractions for all patients using a simultaneous integrated boost technique.

sMRI-modified target volumes were generated using Cho/NAA maps to account for tumor infiltration into the surrounding tissue (13). Cho/NAA thresholds depicting 1.5-, 1.75-, and 2.0-fold increases were segmented, representing absolute Cho/NAA ratios of 0.9–1.2, which have been shown to be pathological (17–19). GTV2 and CTV2 volumes for the sMRI-modified plans were generated as described for the conventional plans. Each Cho/NAA segmentation was merged with the conventional CTV2 to create an “sMRI_CTV2” to more sensitively account for microscopic disease (13). An additional 0.3-cm margin was added to create the “sMRI_PTV2.” For replanning, each sMRI_CTV2 was merged with CTV1 to create “sMRI_CTV1,” to which a 0.3-cm margin was added to generate “sMRI_PTV1.”

### Data Analysis, Replanning, and Recurrence Evaluation

Each Cho/NAA segmentation and target volume was imported to Matlab (The MathWorks, Inc.) as a Digital Imaging and Communications in Medicine (DICOM)-RT structure with coregistered CE-T1w images and rasterized into the CE-T1w image space. For each threshold, the sMRI_CTV2 and CTV2 were compared in terms of absolute volume and spatial overlap using the Dice similarity coefficient (DICE) (20, 21). In addition, the percent volume of each Cho/NAA segmentation extending beyond the CTV1, CTV2, and respective 100% prescription isodose lines (IDLs) was calculated.

sMRI-modified RT plans were replanned in the Eclipse Treatment Planning System (Varian Medical Systems) by medical dosimetrists using sMRI-modified target volumes to evaluate target coverage and organ-at-risk dose. The planning goal was set for 100% of the dose to cover 95% of the target volume, while limiting the maximum dose to the brainstem, optic chiasm, and optic nerves to <54 Gy. A maximum point dosage of 60 Gy to the brainstem with no more than 10% above 54 Gy was a hard constraint. Coverage of the PTV60 to 90% was allowable in cases where the brainstem and tumor were in close proximity.

Follow-up imaging was analyzed to determine the coverage of contrast-enhancing recurrent tumor by conventional and sMRI-modified plans. Follow-up MRI scans acquired at 2- to 3-month intervals were evaluated by radiation oncologists for tumor progression using the RANO criteria (22). In patients with progression (confirmed with subsequent imaging or biopsy), recurrent tumor was segmented, confirmed by a neuroradiologist, and compared with the treatment plans to evaluate recurrent tumor coverage.

### Statistical Methods

Statistical analyses were performed with the Matlab Statistics and Machine Learning Toolbox with significance set at *P* ≤ .05. Differences in conventional and sMRI-modified target volumes and percentage of recurrence outside of CTV2 were evaluated using one-sided paired *t* tests. Target coverage and brainstem doses were evaluated using one-way analysis of variance. Differences in DICE indices were evaluated using one-sided *t* tests for population means (set at 1.0) with unknown variances.

## Results

### Volumetric and Spatial Analysis

**Figure 1.**
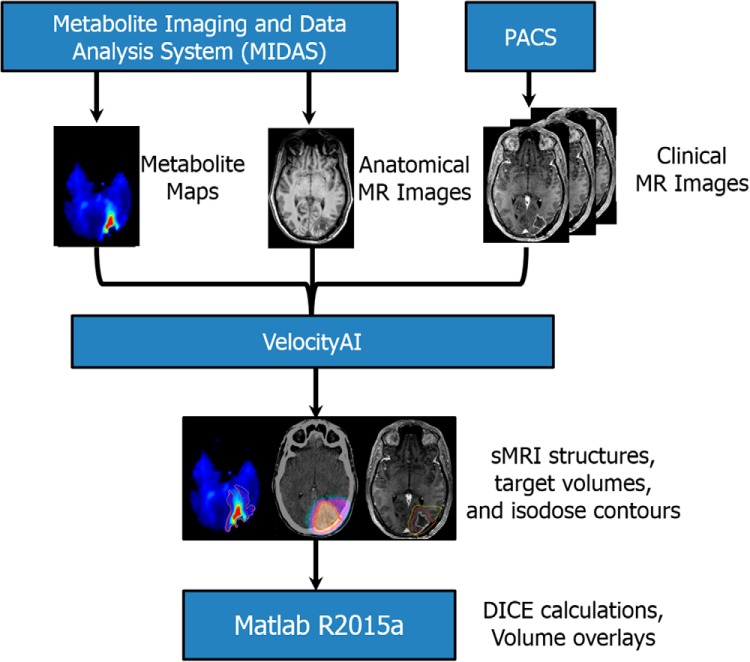
Image processing and analysis pipeline. Spectral processing in Metabolite Imaging and Data Analysis System (MIDAS) includes signal normalization, tissue segmentation, quality evaluation, and registration of metabolite maps to anatomical images. Anatomical, metabolite, and clinical images are imported into VelocityAI for coregistration and radiation therapy (RT) volume contouring. Contralateral normal-appearing white matter is segmented using white matter probability maps to determine fold changes in spectroscopic magnetic resonance imaging (sMRI) metabolites. sMRI maps depicting standardized metabolite changes are then viewed alongside clinical images to assist in target delineation.

**Figure 2.**
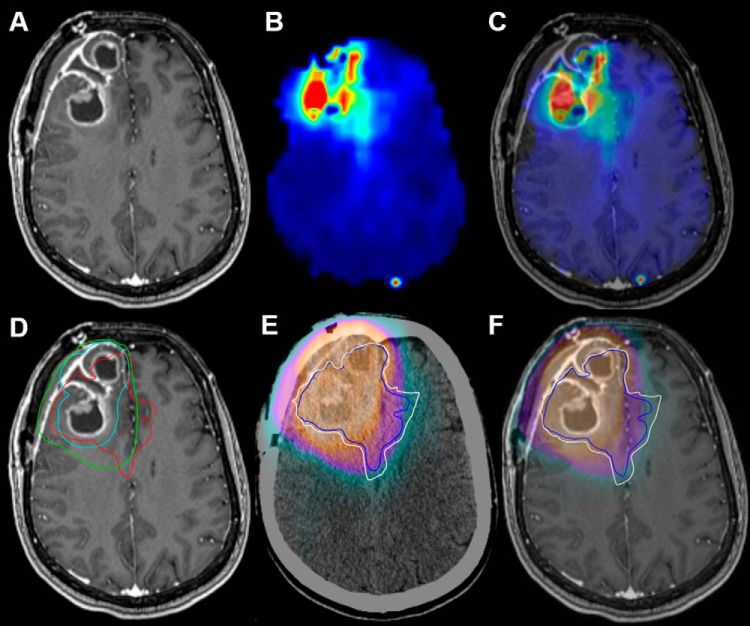
Contrast-enhanced T1-weighted (CE-T1W), choline to N-Acetylaspartate (Cho/NAA) map, and dose fields for a patient with a frontal glioblastoma (GBM) who received standard chemoradiation (patient 3). Cho/NAA abnormalities extended medially and posteriorly from the contrast-enhancing border, implying contralateral infiltration (A–C). A region of 2.0-fold Cho/NAA increase (red contour) falls outside the clinical target volumes (CTVs)2 (turquoise contour) and CTV1 (green contour) near the midline (D). A comparison of the cumulative RT dose with 2.0-fold (blue contour) and 1.75-fold (white contour) Cho/NAA changes shows a large portion of metabolic abnormality falling outside of the region receiving 60 Gy (E and F).

**Figure 3.**
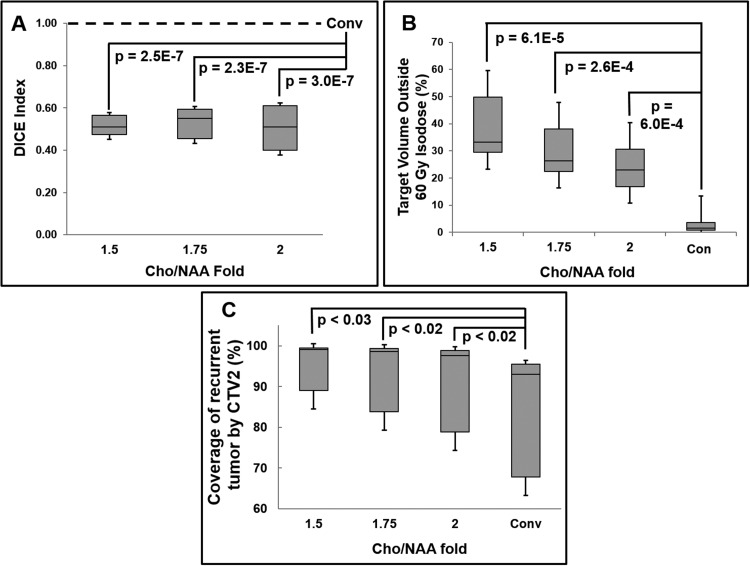
Spatial and volumetric comparison of sMRI abnormalities and plans with those generated using conventional imaging. The spatial overlap for each Cho/NAA threshold with the CTV2 was shown to be significantly different from unity (A), with significant volumes of each threshold falling outside of the 60 Gy IDLs (B). sMRI-modified plans exhibited significantly higher coverage of contrast-enhancing tumor at recurrence than conventional plans (C). DICE and metabolic volumes outside of 60 Gy isodose lines (IDLs) were compared with unity and the planning target volume (PTVs)2 outside of the 60 Gy IDLs for conventional plans, respectively.

Whole-brain sMRI data were obtained from 11 patients between July 2014 and March 2015 (Supplemental Table 1) and processed using the pipeline described above (also shown in Figure 1). Of note, a single patient (patient 8) had an unresectable tumor and did not undergo surgical resection before chemoradiation. An example of MRI, Cho/NAA, and RT dosage plan overlays in a patient with superimposed CTVs and Cho/NAA thresholds can be found in Figure 2. The mean volume percentage increase in sMRI_CTV2 was 74.1%, 42.2%, and 25.1% for Cho/NAA thresholds of 1.5-, 1.75-, and 2.0-fold, respectively (Supplemental Table 2). The 1.5-fold sMRI_CTV2 volume increased significantly, although 1.75- and 2.0-fold sMRI_CTV2s did not. MRI_CTV1 also increased, but to a smaller extent (14.1%, 7.88%, and 5.02% at 1.5-, 1.75-, and 2.0-fold, respectively) and not significantly (*P* = .96). Significantly decreased spatial overlap was noted between the conventional CTV and all Cho/NAA segmentations with mean DICE scores of 0.51, 0.53, and 0.51 (1.5-, 1.75-, and 2.0-fold, respectively; Figure 3A; Supplemental Table 2). The mean Cho/NAA abnormality extending outside of the 100% IDL of PTV1 and PTV2 was 12.4 cm^3^ and 41.1 cm^3^, 5.36 cm^3^ and 22.2 cm^3^, and 2.37 cm^3^ and 12.7 cm^3^ for 1.5-, 1.75-, and 2.0-fold thresholds, Supplemental Table 3). The percentage of sMRI volume extending beyond the 60 Gy IDL was significantly increased for each Cho/NAA threshold (Figure 3B); however, for the 51 Gy IDL, only the 1.5-fold threshold was significant (*P* = .03).

### Replanning

Table 1 details target coverage and maximum brainstem dose for each patient. Replanning was successful and clinically acceptable for all patients at each Cho/NAA threshold. In all plans, the maximum dosage to the optic nerves and chiasm met specified constraints. The average target volume coverage for sMRI_PTV 1 and 2 was not significantly different from conventional plans (*P* = .172 and 0.110, respectively). Only 2 patients' revised plans did not closely meet the desired target coverage (for PTV2 [60 Gy]) because of brainstem proximity. However, PTV2 coverage in the conventional plan was not achieved in either case. The maximum brainstem dose was significantly higher in sMRI-modified plans, likely because of the substantial increase in the dosage for patients 3 and 11 (13.0 and 7.3 Gy and 59.8 and 58.4 Gy, respectively). However, the brainstem received <60 Gy in all cases.

**Table 1.** Target Coverage and Brainstem Dosages for Conventional and sMRI-modified RT Plans

	Patient	1	2	3	4	5	6	7	8	9	10	11	Median
Target volume coverage (%) of PTV1 by prescription dosage (54 Gy)	Conv_PTV1	98.0	98.5	100.0	99.9	99.5	94.6	97.9	99.9	97.5	98.8	99.9	98.80
	sMRI-PTV1 (1.50-fold)	99.5	99.9	100.0	99.9	100.0	98.7	99.1	99.4	98.3	100.0	99.9	99.90
	sMRI-PTV1 (1.75-fold)	99.9	100.0	100.0	99.9	99.9	98.7	99.1	96.7	98.1	100.0	99.9	99.90
	sMRI-PTV1 (2.0-fold)	99.9	100.0	100.0	99.9	99.7	97.8	98.8	99.2	99.1	99.7	99.9	99.70
Target volume coverage (%) of PTV2 by prescription dosage (60 Gy)	Conv_PTV2	98.0	96.8	97.9	86.2	98.9	96.5	98.9	98.7	89.5	99.9	99.4	98.00
	sMRI-PTV2 (1.50-fold)	91.0	93.5	95.5	83.0	94.7	94.5	95.0	94.6	80.0	95.0	95.0	94.60
	sMRI-PTV2 (1.75-fold)	95.0	94.9	95.0	84.0	95.4	94.3	95.0	95.0	85.1	95.1	94.1	95.00
	sMRI-PTV2 (2.0-fold)	96.0	95.0	95.0	86.0	95.9	94.0	95.0	94.9	91.3	95.5	94.8	95.00
Brainstem maximum dosage (Gy)	Conv_PTV1	53.5	55.0	13.0	59.3	52.8	53.9	59.4	58.3	58.0	52.9	7.3	53.90
	sMRI-PTV1 (1.50-fold)	58.9	59.9	59.8	57.9	57.8	59.4	58.7	59.6	58.3	58.8	58.0	58.80
	sMRI-PTV1 (1.75-fold)	58.7	58.8	58.1	58.9	58.1	59.2	59.0	59.4	59.2	58.4	58.4	58.80
	sMRI-PTV1 (2.0-fold)	58.7	59.2	55.4	58.2	57.8	58.7	58.6	58.9	59.3	58.7	58.2	58.70

Abbreviations: PTV1, planning target volume 1 (corresponding to PTV51 or PTV54); PTV2, planning target volume 2 (corresponding to PTV60); Conv, conventional.

### Recurrent Tumor Coverage Analysis

**Figure 4.**
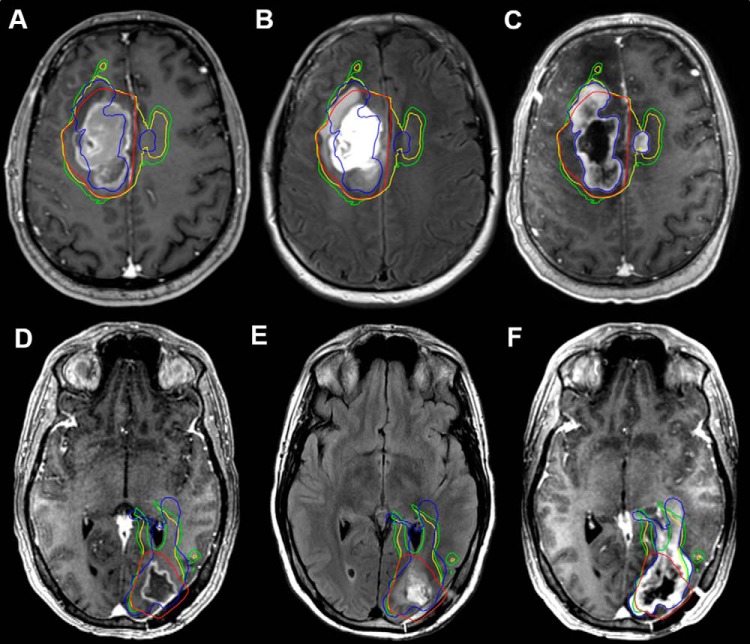
PreRT CE-T1W, PreRT T2-weighted/FLAIR, and recurrence CE-T1W images with conventional, 1.75-fold, and 2.0-fold CTVs (red, green, and yellow contours, respectively) for patients 4 (A–C) and 11 (D–F). Regions of tumor recurrence (blue contour) anterior and contralateral to contrast enhancement in patient 4 (A) and passing toward the ventricle in patient 11 (D) were not encompassed by the CTV60. The fusion of 1.75- and 2.0-fold Cho/NAA abnormalities with the CTV60 resulted in a target covering a significantly larger proportion of the recurrence.

Median follow-up time was 11.7 months, during which tumor recurrence was observed in 7 of 11 patients, with a median time-to-recurrence of 3.3 months (interquartile range, 2.48 mo; range, 0.97–8.8 mo). Preliminary analysis of recurrence location shows that in 6 of 7 patients, sMRI-modified CTVs encompassed regions of contrast-enhancing tumor at recurrence that was not targeted with conventional plans. In these patients, the coverage of contrast enhancement at recurrence was significantly larger for 1.5-, 1.75-, and 2.0-fold Cho/NAA sMRI_CTVs on average (92.4%, 90.5%, and 88.6%, respectively) than for conventional CTV (82.5%) (Figure 3C). Representative examples can be found in Figure 4.

**Figure 5.**
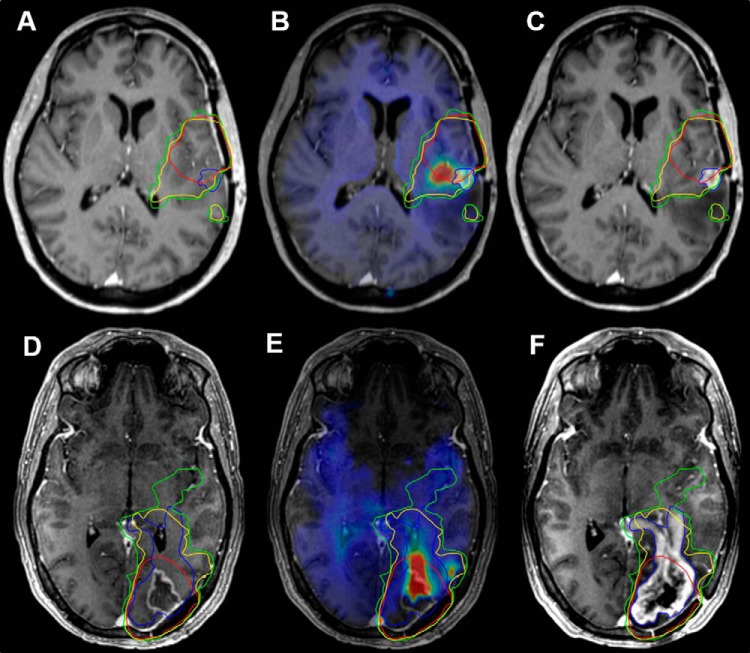
PreRT CE-T1W, PreRT Cho/NAA map, and recurrence CE-T1W images with 60 Gy IDLs from conventional, 1.75-fold, and 2.0-fold CTVs' plans (red, green, and yellow contours, respectively) for patients 9 (A–C) and 11 (D–F). The 60 Gy IDLs from the conventional RT plans cover 84.1% and 68.1% of the contrast-enhancing tumor at the time of recurrence (blue contour) in patients 9 and 11, respectively. However, 60 Gy IDLs generated with 1.75-fold (green contours) and 2.00-fold (yellow contours) Cho/NAA thresholds cover a greater proportion of the recurrent tumor (96.1% and 92.4%, in patient 9; 97.6% and 95.3% in patient 11). sMRI-modified 60 Gy IDLs reflect PTV2s generated by a 0.3 cm expansion to the union of the CTV2 with a Cho/NAA-fold threshold.

More than 95% of recurrent tumors fell within the 60 Gy IDL of the original plan in 5 out of 7 patients. However, patients 9 and 11 (Figure 5) exhibited a substantial proportion of contrast enhancement outside of the 60 Gy (15.8% and 31.9%, respectively) and 57 Gy IDLs (4.4% and 28.1%, respectively). If contrast enhancement at recurrence was known and considered as a target volume in planning, 95% of the recurrence would not receive 60 Gy and 100% of the recurrence would not receive 57 Gy using the conventional plan in either case. However, replanning with sMRI_CTVs increased the 60 and 57 Gy coverage of recurrent tumor to meet these planning objectives. Using 1.5-, 1.75-, and 2.0-fold Cho/NAA sMRI_CTVs, for patient 9, 96.1%, 92.4%, and 87.4% of recurrent tumors received 60 Gy, whereas 99.1%, 98.9%, and 97.1% tumors received 57 Gy, respectively. Similarly, for patient 11, 97.6%, 95.3%, and 92.5% of recurrent tumors received 60 Gy, whereas 99.0%, 97.3%, and 96.4% received 57 Gy, respectively.

## Discussion

Several groups have reported that spectroscopy identifies regions of tumor infiltration beyond contrast enhancement, even within edema (23–27). In addition, a study recently published by our group found Cho/NAA to be highly correlated with the presence of neoplastic cells in patients with GBM receiving surgery (28). As this ratio comprises markers describing membrane lipid biosynthesis (eg, choline) and neuronal integrity (eg, N-Acetylaspartate), peritumoral Cho/NAA elevations indicate the presence of a highly proliferative, non-neuronal cell population that is highly suggestive of tumor infiltration. In this work, we describe a clinical workflow that enables the incorporation of the Cho/NAA ratio from sMRI into RT planning to improve target volume definition in patients with GBM. We also show that the addition of this metabolic information can significantly alter RT treatment plans in such a way that may improve local control.

MRSI has previously been integrated in RT planning; however, these reports exhibit a number of technical limitations (3, 8–12, 29). First, the partial volume effects and limited coverage of the low-resolution, limited field-of-view techniques used severely limit the use of MRSI clinically. Second, none of these studies uses systematic methods for normalizing spectroscopic signal, standardizing abnormalities, and transferring metabolite volumes to RT planning software for target modification alongside conventional images. The lack of such a processing protocol not only makes the use of spectroscopy in RT planning subjective, as clinicians must compare arrayed spectra qualitatively, but also time-consuming and labor-intensive. To overcome these limitations, we used a higher-resolution, whole-brain spectroscopic sequence that gives nominal voxel size of 4.4 × 4.4 × 5.6 mm^3^, ultimately increasing the utility of spectroscopic data. Furthermore, we established a pipeline to quickly process and integrate normalized sMRI maps into the clinical RT planning workflow.

The addition of Cho/NAA to RT planning considerably modified target volumes and described metabolic abnormalities outside of conventional dose regions. For all but the 1.5-fold threshold, Cho/NAA and conventional CTVs were similar. However, DICE scores were significantly lower for each Cho/NAA threshold, suggesting significant spatial differences from associated CTVs. Taken together, these data suggest that the shape and/or location of the target are/is changing without adding a substantial brain volume receiving RT. In addition, the substantial Cho/NAA abnormality beyond the 60 Gy IDL suggests that metabolically active disease does not receive appropriate coverage in conventional RT plans. Similar results were noted in a 2003 report (10), where 21 cm^3^ of spectroscopic abnormality, on average, fell beyond contrast enhancement in patients with high-grade glioma. Similarly, in a recent study, one-third of the patients receiving RT had metabolically active tumor outside of the CTV60 threshold, many of which expanded beyond 51 Gy and/or 54 Gy IDLs (12). Because the Cho/NAA ratio has been shown to be strongly associated with tumor infiltration in patients with GBM, expansion of the 60 Gy target volume to cover regions that exhibit Cho/NAA elevations may improve local control (13, 26, 27).

The addition of Cho/NAA abnormalities to RT planning resulted in a higher coverage of recurrent contrast-enhancing tumor compared with conventional plans in all cases. In 2 cases, contrast enhancement at recurrence that was Cho/NAA abnormal before treatment fell beyond the 60 Gy IDLs in the conventional plan. Using sMRI-modified plans, expanded 60 Gy IDLs encompassed up to 97.6% of the recurrent tumor in these patients while respecting coverage and organ-at-risk constraints. Targeting plans created from metabolically active regions instead of conventional plans may delay recurrence and should be investigated in larger, prospective trials.

Some weaknesses of this study warrant consideration. Although this pipeline was executed within an RT planning framework, spectral and volumetric processing could be improved. For example, magnetic susceptibility obscures the spectral profile near tissue boundaries, resulting in artifacts. These artifacts can obscure tumor-related abnormalities, making it difficult to accurately define the metabolic margins. Errors in lipid suppression can also yield artifacts; however, none was observed in regions that were used for RT planning. At the signal acquisition level, sophisticated magnetic field shimming protocols are being developed to account for susceptibility changes and attenuate spectral artifacts (30). In addition, we are developing a spectral quality filter to remove spectral artifacts from metabolite maps using machine-learning techniques. Finally, we examined only 3 Cho/NAA-fold levels, whereas the identification of an optimal cutoff for use in RT planning may require evaluating finer Cho/NAA intervals. As such, a sensitivity study using a large number of Cho/NAA thresholds is currently underway to identify the ideal threshold for a future RT dose expansion and escalation study.

## Conclusions

Using a recently developed pipeline, the integration of high-resolution, whole-brain sMRI into RT planning is feasible and resulted in considerably modified RT target volumes that allow the specific targeting of tumor-infiltrated tissue while adhering to normal planning objectives. Such an addition would represent a paradigm shift in the field of image-guided RT away from targeting surrogate markers using tracer-based imaging techniques to targeting imaging biomarkers that describe native biochemical processes. Given the apparent effectiveness of sMRI in identifying regions at high risk for recurrence, this technique shows promise for improving the outcomes for patients with GBM and should be evaluated in larger, prospective clinical trials.

### Supplemental Materials

Supplemental Table 1–3:

## References

[1] Burger PC, Dubois PJ, Schold SC Jr., Smith KR Jr., Odom GL, Crafts DC, Giangaspero F Computerized tomographic and pathologic studies of the untreated, quiescent, and recurrent glioblastoma multiforme. J Neurosurg. 1983;58(2):159–169. 6294260 10.3171/jns.1983.58.2.0159

[2] McKnight TR, von dem Bussche MH, Vigneron DB, Lu Y, Berger MS, McDermott MW, Dillon WP, Graves EE, Pirzkall A, Nelson SJ Histopathological validation of a three-dimensional magnetic resonance spectroscopy index as a predictor of tumor presence. J Neurosurg. 2002;97(4):794–802. 12405365 10.3171/jns.2002.97.4.0794

[3] Narayana A, Chang J, Thakur S, Huang W, Karimi S, Hou B, Kowalski A, Perera G, Holodny A, Gutin PH Use of MR spectroscopy and functional imaging in the treatment planning of gliomas. Br J Radiol. 2007;80(953):347–354. 17068012 10.1259/bjr/65349468

[4] Farace P, Giri MG, Meliado G, Amelio D, Widesott L, Ricciardi GK, Dall'Oglio S, Rizzotti A, Sbarbati A, Beltramello A, Maluta S, Amichetti M Clinical target volume delineation in glioblastomas: pre-operative versus post-operative/pre-radiotherapy MRI. Br J Radiol. 2011;84(999):271–278. 21045069 10.1259/bjr/10315979 PMC3473876

[5] Burger PC, Heinz ER, Shibata T, Kleihues P Topographic anatomy and CT correlations in the untreated glioblastoma multiforme. J Neurosurg. 1988;68(5):698–704. 2833587 10.3171/jns.1988.68.5.0698

[6] Cao Y, Sundgren PC, Tsien CI, Chenevert TT, Junck L Physiologic and metabolic magnetic resonance imaging in gliomas. J Clin Oncol. 2006;24(8):1228–1235. 16525177 10.1200/JCO.2005.04.7233

[7] Nelson SJ Multivoxel magnetic resonance spectroscopy of brain tumors. Mol Cancer Ther. 2003;2(5):497–507. 12748312

[8] Nelson SJ, Graves E, Pirzkall A, Li X, Antiniw Chan A, Vigneron DB, McKnight TR In vivo molecular imaging for planning radiation therapy of gliomas: an application of 1H MRSI. J Magn Reson Imaging. 2002;16(4):464–476. 12353260 10.1002/jmri.10183

[9] Einstein DB, Wessels B, Bangert B, Fu P, Nelson AD, Cohen M, Sagar S, Lewin J, Sloan A, Zheng Y, Williams J, Colussi V, Vinkler R, Maciunas R Phase II trial of radiosurgery to magnetic resonance spectroscopy-defined high-risk tumor volumes in patients with glioblastoma multiforme. Int J Radiat Oncol Biol Phys. 2012;84(3):668–674. 22445005 10.1016/j.ijrobp.2012.01.020 PMC4334318

[10] Pirzkall A, Li X, Oh J, Chang S, Berger MS, Larson DA, Verhey LJ, Dillon WP, Nelson SJ 3D MRSI for resected high-grade gliomas before RT: tumor extent according to metabolic activity in relation to MRI. Int J Radiat Oncol Biol Phys. 2004;59(1):126–137. 15093908 10.1016/j.ijrobp.2003.08.023

[11] Ken S, Vieillevigne L, Franceries X, Simon L, Supper C, Lotterie JA, Filleron T, Lubrano V, Berry I, Cassol E, Delannes M, Celsis P, Cohen-Jonathan EM, Laprie A Integration method of 3D MR spectroscopy into treatment planning system for glioblastoma IMRT dose painting with integrated simultaneous boost. Radiat Oncol. 2013;8:1. 23280007 10.1186/1748-717X-8-1 PMC3552736

[12] Parra NA, Maudsley AA, Gupta RK, Ishkanian F, Huang K, Walker GR, Padgett K, Roy B, Panoff J, Markoe A, Stoyanova R Volumetric spectroscopic imaging of glioblastoma multiforme radiation treatment volumes. Int J Radiat Oncol Biol Phys. 2014;90(2):376–84. 25066215 10.1016/j.ijrobp.2014.03.049 PMC4346247

[13] Cordova JS, Shu HG, Liang Z, Gurbani SS, Cooper LA, Holder CA, Olson JJ, Kairdolf B, Schreibmann E, Neill SG, Hadjipanayis CG, Shim H Whole-brain spectroscopic MRI biomarkers identify infiltrating margins in glioblastoma patients. Neuro Oncol. 2016;18(8):1180–1189. 26984746 10.1093/neuonc/now036 PMC4933486

[14] Maudsley AA, Domenig C, Govind V, Darkazanli A, Studholme C, Arheart K, Bloomer C Mapping of brain metabolite distributions by volumetric proton MR spectroscopic imaging (MRSI). Magn Reson Med. 2009;61(3):548–559. 19111009 10.1002/mrm.21875 PMC2724718

[15] Maudsley AA, Darkazanli A, Alger JR, Hall LO, Schuff N, Studholme C, Yu Y, Ebel A, Frew A, Goldgof D, Gu Y, Pagare R, Rousseau F, Sivasankaran K, Soher BJ, Weber P, Young K, Zhu X Comprehensive processing, display and analysis for in vivo MR spectroscopic imaging. NMR Biomed. 2006;19(4):492–503. 16763967 10.1002/nbm.1025 PMC2673915

[16] Cordova JS, Gurbani SS, Olson JJ, Liang Z, Cooper LAD, Shu HG, Schreibmann E, Neill SG, Hadjipanayis CG, Holder CA, Shim H A systematic pipeline for the objective comparison of whole-brain spectroscopic MRI with histology in biopsy specimens from grade III glioma. Tomography. 2016;2(2):106–116. 27489883 10.18383/j.tom.2016.00136 PMC4968944

[17] Guo J, Yao C, Chen H, Zhuang D, Tang W, Ren G, Wang Y, Wu J, Huang F, Zhou L The relationship between Cho/NAA and glioma metabolism: implementation for margin delineation of cerebral gliomas. Acta Neurochir (Wien). 2012;154(8):1361–1370; discussion 70. 22729482 10.1007/s00701-012-1418-x PMC3407558

[18] Widhalm G, Krssak M, Minchev G, Wohrer A, Traub-Weidinger T, Czech T, Asenbaum S, Marosi C, Knosp E, Hainfellner JA, Prayer D, Wolfsberger S Value of 1H-magnetic resonance spectroscopy chemical shift imaging for detection of anaplastic foci in diffusely infiltrating gliomas with non-significant contrast-enhancement. J Neurol Neurosurg Psychiatry. 2011;82(5):512–520. 20971752 10.1136/jnnp.2010.205229

[19] Croteau D, Scarpace L, Hearshen D, Gutierrez J, Fisher JL, Rock JP, Mikkelsen T Correlation between magnetic resonance spectroscopy imaging and image-guided biopsies: semiquantitative and qualitative histopathological analyses of patients with untreated glioma. Neurosurgery. 2001;49(4):823–829. 11564242 10.1097/00006123-200110000-00008

[20] Chang HH, Zhuang AH, Valentino DJ, Chu WC Performance measure characterization for evaluating neuroimage segmentation algorithms. Neuroimage. 2009;47(1):122–135. 19345740 10.1016/j.neuroimage.2009.03.068

[21] Dice LR Measures of the amount of ecologic association between species. Ecology. 1945;26(3):297–302.

[22] Wen PY, Macdonald DR, Reardon DA, Cloughesy TF, Sorensen AG, Galanis E, Degroot J, Wick W, Gilbert MR, Lassman AB, Tsien C, Mikkelsen T, Wong ET, Chamberlain MC, Stupp R, Lamborn KR, Vogelbaum MA, van den Bent MJ, Chang SM Updated response assessment criteria for high-grade gliomas: response assessment in neuro-oncology working group. J Clin Oncol. 2010;28(11):1963–1972. 20231676 10.1200/JCO.2009.26.3541

[23] Di Costanzo A, Scarabino T, Trojsi F, Giannatempo GM, Popolizio T, Catapano D, Bonavita S, Maggialetti N, Tosetti M, Salvolini U, d'Angelo VA, Tedeschi G Multiparametric 3T MR approach to the assessment of cerebral gliomas: tumor extent and malignancy. Neuroradiology. 2006;48(9):622–631. 16752135 10.1007/s00234-006-0102-3

[24] Di Costanzo A, Scarabino T, Trojsi F, Popolizio T, Catapano D, Giannatempo GM, Bonavita S, Portaluri M, Tosetti M, d'Angelo VA, Salvolini U, Tedeschi G Proton MR spectroscopy of cerebral gliomas at 3 T: spatial heterogeneity, and tumour grade and extent. Eur Radiol. 2008;18(8):1727–1735. 18389246 10.1007/s00330-008-0938-5

[25] Law M MR spectroscopy of brain tumors. Top Magn Reson Imaging. 2004;15(5):291–313. 15627004 10.1097/00002142-200410000-00003

[26] Stadlbauer A, Buchfelder M, Doelken MT, Hammen T, Ganslandt O Magnetic resonance spectroscopic imaging for visualization of the infiltration zone of glioma. Cent Eur Neurosurg. 2011;72(2):63–69. 20635312 10.1055/s-0030-1253410

[27] Stadlbauer A, Nimsky C, Buslei R, Pinker K, Gruber S, Hammen T, Buchfelder M, Ganslandt O Proton magnetic resonance spectroscopic imaging in the border zone of gliomas: correlation of metabolic and histological changes at low tumor infiltration–initial results. Invest Radiol. 2007;42(4):218–223. 17351427 10.1097/01.rli.0000255812.61435.67

[28] Cordova JS, Shu HG, Liang Z, Gurbani SS, Cooper LAD, Holder CA, Olson JJ, Kairdolf B, Schreibmann E, Neill S, Hadjipanayis CG, Shim H Whole-brain, spectroscopic MRI biomarkers identify infiltrating margins in glioblastoma patients. Neuro Oncol. 2016;18(8):1180–1189. 26984746 10.1093/neuonc/now036 PMC4933486

[29] Chang J, Thakur SB, Huang W, Narayana A Magnetic resonance spectroscopy imaging (MRSI) and brain functional magnetic resonance imaging (fMRI) for radiotherapy treatment planning of glioma. Technol Cancer Res Treat. 2008;7(5):349–362. 18783284

[30] Stockmann JP, Witzel T, Keil B, Polimeni JR, Mareyam A, LaPierre C, Setsompop K, Wald LL A 32-channel combined RF and B0 shim array for 3T brain imaging. Magn Reson Med. 2016;75(1):441–451. 25689977 10.1002/mrm.25587 PMC4771493

