# Supplementary material for: Simulating the Effect of Spectroscopic MRI as a Metric for Radiation Therapy Planning in Patients with Glioblastoma
Source: Tomography. 2016 Dec;2(4):366–73. doi: 10.18383/j.tom.2016.00187 (PMC5241103; doi:10.18383/j.tom.2016.00187)
Supplement: Supplemental Table 1–3: [file tom-00187-16-s001.pdf]

Supplemental Data

Simulating the Effect of Spectroscopic MRI as a Metric for Radiation Therapy Planning in Patients with Glioblastoma

*J. Scott Cordova[1], *Shravan Kandula[2, 3], Saumya Gurbani[1,6], Jim Zhong MD[2], Mital Tejani[2], Oluwatosin Kayode[2], Kirtesh Patel[2], Roshan Prabhu[4], Eduard Schreibmann[2], Ian Crocker[2, 5], Chad A. Holder[1], Hyunsuk Shim[1, 5, 6], and Hui-Kuo Shu[2, 5]

[1]Department of Radiology and Imaging Sciences, Emory University School of Medicine, 1648 Pierce Dr NE, Atlanta, GA, USA; [2]Department of Radiation Oncology, Emory University School of Medicine, 1648 Pierce Dr NE, Atlanta, GA, USA; [3]Florida Hospital Medical Group, Radiation Oncology Associates, 2501 N Orange Avenue, Orlando, FL, USA; [4]Southeast Radiation Oncology Group, Levine Cancer Institute, 1021 Morehead Medical Dr Charlotte, NC, USA; [5]Winship Cancer Institute, 1365-C Clifton Rd NE Atlanta, GA, USA; [6]Department of Biomedical Engineering, Georgia Institute of Technology, Atlanta, GA, USA.

*Authors contributed equally to this work.

Journal: Tomography

Corresponding Authors
Hyunsuk Shim, Ph.D.
Department of Radiology
Emory University School of Medicine
1701 Uppergate Drive, C5018, Atlanta, GA 30322
Telephone: (404) 778-4564
E-mail: hshim@emory.edu

Hui-Kuo Shu, MD, PhD
Department of Radiation Oncology
Emory University School of Medicine
1365 Clifton Rd, Atlanta, GA 30322.
Telephone: (404) 778-3473
E-mail: hgshu@emory.edu

Supplementary Table 1. Patient, tumor, and target characteristics

Patient	Age (yrs)	Tumor location	IDH	MGMT	Surgical Outcome	GTV1 (cm^3)	GTV2 (cm^3)
1	64	Right parieto-temporal	wt	unmethylated	GTR	154.6	46.4
2	62	Left temporo-occipital	wt	unmethylated	STR	175.9	48.4
3	56	Right frontal	wt	unmethylated	STR	109.2	43.4
4	43	Right frontal	wt	unmethylated	STR	254.0	78.8
5	83	Right parietal	wt	unmethylated	STR	292.9	37.3
6	46	Left parietal	wt	N/A	STR	72.1	34.8
7	45	Left parieto-temporal	wt	unmethylated	GTR	89.7	14.8
8	60	Right parieto-temporal	wt	methylated	Biopsy	154.6	35.1
9	46	Left temporal	mt	methylated	STR	204.0	35.3
10	73	Left temporal	wt	methylated	STR	74.7	24.0
11	41	Left occipital	wt	methylated	GTR	53.1	17.4

Abbreviations: wt – wild type. GTV1 – Gross target volume 1 (corresponding to T2-FLAIR abnormality). GTV2 – Gross target volume 2 (corresponding to the resection cavity and residual contrast-enhancing tumor). GTR – gross total resection (≤ 1 cm^3 contrast-enhancing tumor on postoperative MRI). STR – subtotal resection (≥ 1 cm^3 contrast-enhancing tumor on postoperative MRI).

Supplementary Table 2. Volumetric and Spatial Data

	Cho/NAA – 1.5-fold							
**Patient**	**CTV1 (cm^3)**	**sMRI_CTV1 (cm^3)**	**%volume difference**	**DICE**	**CTV2 (cm^3)**	**sMRI_CTV2 (cm^3)**	**%volume difference**	**DICE**
1	276.5	287.0	3.8	0.60	95.7	148.5	55.3	0.67
2	298.3	306.5	2.7	0.53	93.1	153.6	70.0	0.54
3	187.2	247.1	32.0	0.62	90.9	191.7	111.1	0.50
4	289.7	295.1	1.86	0.34	103.8	123.6	19.1	0.50
5	415.7	449.6	8.2	0.47	148.6	219.7	47.8	0.58
6	417.2	462.9	11.0	0.29	91.4	127.1	39.0	0.45
7	178.7	184.9	3.5	0.42	43.3	72.8	68.2	0.51
8	343.9	349.0	1.5	0.53	90.3	134.9	49.3	0.75
9	347.1	352.1	1.4	0.37	88.1	123.2	39.8	0.55
10	138.5	163.4	18.0	0.46	44.9	89.4	99.1	0.32
11	111.1	189.8	70.8	0.38	45.6	144.2	216.1	0.21
Mean	273.1	298.9	14.1	0.46	85.1	139.0	74.1	0.51
SD	106.4	100.5	21.0	0.102	31.0	41.4	54.1	0.142

	Cho/NAA – 1.75-fold							
**Patient**	**CTV1 (cm^3)**	**sMRI_CTV1 (cm^3)**	**%volume difference**	**DICE**	**CTV2 (cm^3)**	**sMRI_CTV2 (cm^3)**	**%volume difference**	**DICE**
1	276.5	283.0	2.4	0.53	95.7	131.9	37.8	0.69
2	298.3	302.3	1.3	0.44	93.1	132.0	41.7	0.55
3	187.2	216.4	15.6	0.62	90.9	153.1	68.5	0.57
4	289.7	294.3	1.6	0.28	103.8	114.0	9.8	0.50
5	415.7	432.9	4.1	0.41	148.6	186.2	25.3	0.62
6	417.2	462.8	10.9	0.19	91.4	108.0	18.1	0.41
7	178.7	183.0	2.4	0.35	43.3	63.5	46.7	0.52
8	343.9	347.7	1.1	0.45	90.3	115.5	27.8	0.79
9	347.1	350.0	0.8	0.29	88.1	106.1	20.4	0.56
10	138.5	153.5	10.8	0.36	44.9	69.5	54.7	0.34
11	111.1	150.8	35.7	0.34	45.6	97.2	113.1	0.26
Mean	273.1	288.8	7.88	0.39	85.1	116.1	42.2	0.53
SD	106.4	106.0	10.5	0.115	31.0	35.0	29.2	0.144

	Cho/NAA – 2.0-fold							
**Patient**	**CTV1 (cm^3)**	**sMRI_CTV1 (cm^3)**	**%volume difference**	**DICE**	**CTV2 (cm^3)**	**sMRI_CTV2 (cm^3)**	**%volume difference**	**DICE**
1	276.5	281.3	1.7	0.48	95.7	122.5	28.1	0.68
2	298.3	300.5	0.7	0.38	93.1	120.0	28.7	0.54
3	187.2	202.8	8.3	0.57	90.9	128.9	41.9	0.60
4	289.7	293.7	1.4	0.23	103.8	109.9	5.9	0.46
5	415.7	425.1	2.3	0.35	148.6	167.4	12.7	0.62
6	417.2	462.8	10.9	0.14	91.4	100.9	10.3	0.34
7	178.7	181.6	1.6	0.30	43.3	57.3	32.4	0.51
8	343.9	346.3	0.7	0.39	90.3	105.1	16.4	0.79
9	347.1	349.6	0.7	0.20	88.1	96.0	8.9	0.50
10	138.5	150.1	8.4	0.27	44.9	59.6	32.6	0.31
11	111.1	131.7	18.5	0.29	45.6	72.2	58.2	0.31
Mean	273.1	284.1	5.02	0.33	85.1	103.6	25.1	0.51
SD	106.4	109.0	5.81	0.119	31.0	32.4	16.1	0.148

Abbreviations: CTV – clinical target volume based on CT and MRI findings; sMRI_CTV – clinical target volume consisting of union of sMRI contour and clinical target volume based on CT and MRI findings; cm^3 – volume in cubic centimeters; DICE - dice similarity coefficient between sMRI contour alone and respective CTV1 or CTV2.

Supplementary Table 3. Cho/NAA contour extending outside of prescription isodoses

Patient	1.5-fold Cho/NAA Contour			
	Volume outside of 100% IDL of PTV1 (cm^3)	% Volume outside of 100% IDL of PTV1	Volume outside of 100% IDL of PTV2(cm^3)	% Volume outside of 100% IDL of PTV2
1	2.8	2.2	36.5	28.4
2	4.6	4.0	45.5	38.9
3	39.9	24.2	81.5	49.6
4	0.4	0.7	20.4	33.3
5	10.5	6.5	49.2	30.5
6	0.0	0.0	36.4	50.1
7	3.5	6.4	15.3	28.1
8	0.1	0.1	16.4	13.0
9	1.2	1.5	25.9	31.6
10	9.1	14.8	34.8	56.7
11	64.1	55.7	90.1	78.2
Mean	12.4	10.6	41.1	39.9
SD	12.01	16.7	19.7	17.7

Patient	1.75-fold Cho/NAA Contour			
	Volume outside of 100% IDL of PTV1 (cm^3)	% Volume outside of 100% IDL of PTV1	Volume outside of 100% IDL of PTV2(cm^3)	% Volume outside of 100% IDL of PTV2
1	1.5	1.5	24.5	23.0
2	2.2	2.5	28.5	31.8
3	15.9	12.9	45.3	36.7
4	0.1	0.3	12.6	26.3
5	3.2	2.7	22.1	18.3
6	0	0	17.5	39.4
7	2.1	4.9	9.5	22.2
8	0.1	0.1	7.5	7.5
9	0.4	0.7	13.6	22.8
10	3.0	7.7	18.0	46.6
11	30.5	46.0	45.5	68.4
Mean	5.36	7.21	22.2	31.2
SD	4.74	13.5	11.1	16.4

Patient	2.0-fold Cho/NAA Contour			
	Volume outside of 100% IDL of PTV1 (cm^3)	% Volume outside of 100% IDL of PTV1	Volume outside of 100% IDL of PTV2(cm^3)	% Volume outside of 100% IDL of PTV2
1	1.0	1.1	17.2	19.1
2	0.9	0.9	19.8	27.6
3	6.8	6.8	25.6	27.5
4	0.03	0.03	8.9	23.0
5	0.6	0.6	9.0	9.5
6	0	0	10.2	33.6
7	1.2	3.4	6.0	17.9
8	0.01	0.02	4.0	4.7
9	0.03	0.07	6.2	15.7
10	1.5	5.8	10.5	40.9
11	14.0	35.1	22.1	55.4
Mean	2.37	4.89	12.7	25.0
SD	2.04	10.3	6.91	14.5

Abbreviations: IDL, isodose line; PTV1, planning target volume 1 (PTV51 or PTV54); PTV2, planning target volume 2 (PTV60).
